# Children and adolescents deaths from trauma-related causes in a Brazilian City

**DOI:** 10.1186/1749-7922-8-52

**Published:** 2013-12-05

**Authors:** Andrea Melo Alexandre Fraga, Joaquim Murray Bustorff-Silva, Thais Marconi Fernandez, Gustavo Pereira Fraga, Marcelo Conrado Reis, Emilio Carlos Elias Baracat, Raul Coimbra

**Affiliations:** 1 Pediatric Emergency Division, Hospital de Clinicas, University of Campinas, Campinas, SP, Brazil; 2 Division of Pediatric Surgery, Department of Surgery, School of Medical Sciences, University of Campinas (Unicamp), Campinas, SP, Brazil; 3 School of Medical Sciences, University of Campinas (Unicamp), Rua Alexander Fleming, 181, Cidade Universitária “Prof. Zeferino Vaz”, Barão Geraldo, Campinas, SP, Brazil; 4 Division of Trauma Surgery, School of Medical Sciences, University of Campinas (Unicamp), Campinas, SP, Brazil; 5 Pediatric Emergency Division, Department of Pediatrician, School of Medical Sciences, University of Campinas (Unicamp), Campinas, SP, Brazil; 6 The Monroe E. Trout Professor of Surgery, Department of Surgery, Division of Trauma, Surgical Critical Care, and Burns, University of California San Diego, 200 West Arbor Dr, #8896, San Diego, CA 92103-8896, USA

**Keywords:** Wounds, Gunshot, Multiple trauma, Drowning, Brain injuries

## Abstract

**Introduction:**

Injury is the first cause of death worldwide in the population aged 1 to 44. In developed countries, the most common trauma-related injuries resulting in death during childhood are traffic accidents, followed by drowning.

**Methods:**

This retrospective study based on autopsy examinations describes the epidemiology profile of deaths by trauma-related causes in individuals younger than 18 years from 2001 to 2008 in the city of Campinas. The aim is to identify epidemiology changes throughout the years in order to develop strategies of prevention.

**Results:**

There were 2,170 deaths from all causes in children < 18 years old, 530 of which were due to trauma-related causes, with a male predominance of 3.4:1. The age distribution revealed that 76% of deaths occurred in the 10-17 age group. The most predominant trauma cause was firearm injury (47%). Other frequent causes were transport-related injuries (138 cases-26%; pedestrians were struck in 57.2% of these cases) and drowning (55 cases-10.4%). Asphyxia/suffocation was the cause of death in 72% of cases in children < 1 year old; drowning (30.8%) was predominant in the 1-4 age group; transport-related deaths were frequent in the 5-9 age group (56%) and the 10-14 age group (40.4%). Gun-related deaths were predominant (68%) in the 14-17 age group. 51% of deaths occurred at the scene.

**Conclusions:**

There was a predominance of deaths in children and adolescents males, between 15-17 years old, mainly from gun-related homicides, and the frequency has decreased since 2004 after the disarmament statute and the combating of violence.

## Introduction

External causes of injuries are the leading cause of death among children and adolescents worldwide and each year more than 950,000 children under the age of 18 die of an injury [1]. Considering the high incidence and diversity of injury, solving this problem is one of the greatest challenges in the field of public health [1-3].

Brazil is the sixth most populous country in the world with approximately 195 million inhabitants, predominantly young. Blessed with abundant natural recourses, Brazil has the most powerful economy in Latin America and has acquired a strong position worldwide. Brazil is slowly improving several social indicators, but socioeconomic and regional disparities are still large [4]. In 2010, approximately 140,000 people died of external causes, and homicides and traffic related deaths accounted for two thirds of all deaths due to trauma-related causes [5]. In 2007, the homicide rate was 26.8 per 100,000 people and the violence has been associated with alcohol and illicit drug use [4].

The number of published studies in international literature from Brazil related to pediatric and adolescents injuries is small [4,6-8]. Fatal injury rates by age group per 100,000 inhabitants in 2003 were 17.7 in Brazilian children less than 5 years old, 10.7 in the 5-9 age group, 14.8 in the 10-14 age group, and 74.7 in the 15-19 age group. In developed countries, injuries due to motor vehicle accidents are the most common [2,9-11]. This high incidence of transport-related deaths is observed in some developing countries such as China, India and Qatar [12-14].

Campinas is a city in the state of São Paulo with about one million inhabitants and each year there are 80 to 200 deaths from trauma-related causes among children. Although located in the most developed state in Brazil, compared with other countries this incidence is very high [8]. There is a need to develop an understanding of traumatic fatalities in children and adolescents to improve injury prevention strategies.

Developing an appropriate approach towards injury prevention in children depends on the knowledge of the epidemiology of traumatic deaths. The aim of this study is to analyze all fatal injuries from trauma-related causes among children and adolescents under 18 years old of age, occurring between 2001 and 2008 in Campinas, in order to identify age groups at risk, mechanism changes during this time period, and develop strategies to decrease the burden through injury prevention activities.

## Materials and methods

Data from the Mortality Information System operated by Brazil’s Ministry of Health reports 5,620 deaths from trauma-related causes in the city of Campinas in the period from January 1^st^, 2001 to December 31^st^, 2008 [5]. This represents 67 deaths from trauma-related causes per 100,000 inhabitants per year. Regarding the population under 18 years of age, there were 2,170 deaths independent of trauma-related causes. The present study selected 530 medico-legal examinations of individuals < 18 years of age who died from trauma-related causes.

In Brazil, by law, medico-legal autopsies are performed in all cases of sudden, suspicious or external cause related deaths. In Campinas there is only one medical examiner’s office (Medical Legal Institute–IML) that performs autopsies on corpses from different cities. This study included only examinations confirmed as trauma-related and exclusively from the city of Campinas. The data for the causes of death were confirmed by the death certificate registry. The medical examiner is a forensic physician with expertise in investigating injury related deaths.

The study was retrospective and descriptive. Data were collected in a database using Excel for Windows (Microsoft™ Redmond, WA). The ages of children were categorized into five groups: less than 1 year, 1-4 years, 5-9 years, 10-14 years and 15-17 years, in order to correlate with causes and intents of death.

The deaths were grouped by cause: drowning, transport-related (car passengers, pedestrians hit by an automobile or train, bicycles, or motorcycles), asphyxia/suffocation, hanging/strangulation, poisoning, burning, stab wound, firearm, fall, assault/blunt trauma, and others. The deaths were also grouped by intent: homicide, self-inflicted (suicide), and unintentional.

To compare trends of mortality, deaths were grouped into two periods, 2001-2004 and 2005-2008. Locations of death were described as: at the scene, pre-hospital care, and at the hospital. The times of death were classified as: immediate (at the scene), less than 24 hours, or more than 24 hours after the injury.

We analyzed the relationships between age group, cause of injury, intent, location, and time of death. The Chi-square test was used as a non-parametric statistical test and the Cochran-Armitage test of trend was carried out to determine the relationship between mechanisms of trauma deaths throughout the years. The level of p < 0.05 was considered as the cut-off value for significance.

Institutional Review Board approval from the IML and the University of Campinas was obtained.

## Results

**Figure 1:**
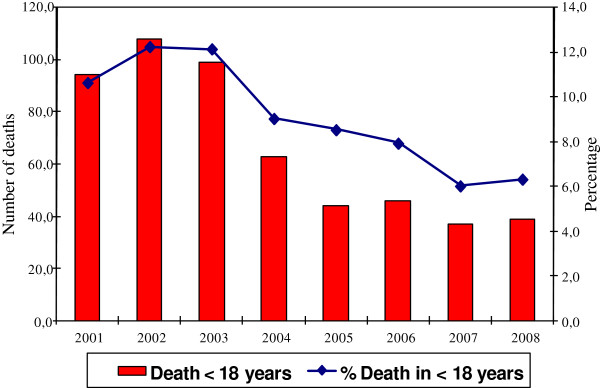
Deaths from external cause and proportion of all deaths among children < 18 years from 2001 to 2008.

Overall, 530 deaths were analyzed. There was a decrease in the number of deaths and proportion of mortality by trauma-related causes in the period 2005-2008 compared to the period 2001-2004 (p < 0.001) (Figure 1).

**Figure 2:**
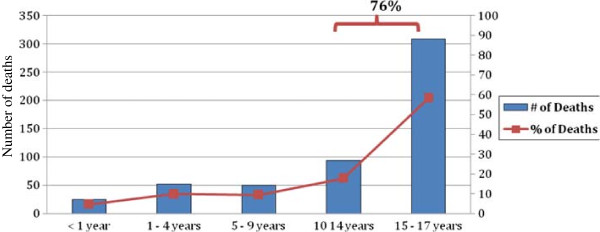
Deaths by age group.

There were 411 males (77.5%) and 119 females (22.5%). The proportion of males to females was 3.4:1 (p < 0.001). 76% of deaths were in children between 10-17 years old (Figure 2).

**Figure 3:**
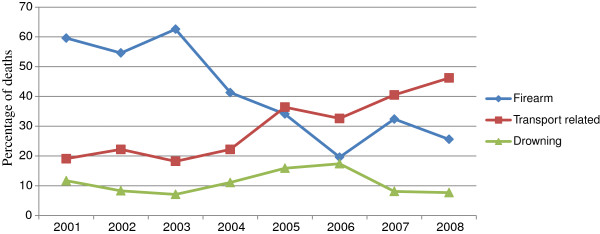
Deaths and most frequent causes of injuries between 2001 and 2008.

Gun-related injury was the most prevalent cause (249 deaths-47%), followed by transport-related injuries (138 deaths-26%) and drowning (55 deaths-10.4%). In the period from 2005 to 2008 the decrease of deaths was a consequence of a marked reduction in gun-related injuries (Figure 3). Using the Cochran-Armitage trend test there was a linear tendency of a decrease in deaths by firearms (p < 0.0001) and an increase in transport-related deaths (p < 0.0001) throughout the years.

Asphyxia/suffocation was the cause of injury in 72% of deaths in group < 1 year; drowning (30.8%) and transport-related injuries (22.8%) were more predominant in the 1-4 age group; transport-related deaths were frequent in the 5-9 age group (56%) and 10-14 age group (40.4%) whilst firearm injuries had the highest frequency in the group 14-17 age group (68%)-Table 1.

**Table 1:** Deaths according to mechanism of injury and age groups

**Mechanism**	**Total**	**<1 year**	**1-4**	**5-9**	**10-14**	**15-17**
	**530**	**25**	**52**	**50**	**94**	**309**
–asphyxia / suffocation	25	18	5	1	-	1
–blunt trauma	14	1	3	1	1	8
–stabb	6	-	-	-	1	5
–drowning	55	1	16	6	14	18
–intoxication	3	1	-	-	-	2
–fall	21	2	5	4	5	5
–burn related	10	-	6	3	-	1
–firearm	249	-	2	4	33	210
–hanging / strangulation	8	1	-	2	2	3
–road traffic related	138	1	15	28	38	56
passenger	44	-	5	9	9	21
pedestrian	77	1	10	18	27	21
train	2	-	-	1	-	1
bicycle	2	-	-	-	1	1
motorcycle	13	-	-	-	1	12
–others	1	-	-	1	-	-

**Figure 4:**
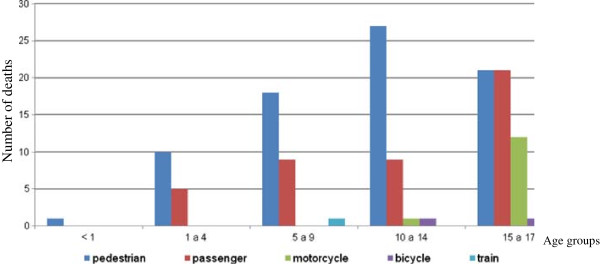
Transport-related deaths by age group.

Pedestrian strike was the cause of injury in 57.2% of transport-related deaths. Two children (9 and 16 years old) were hit by a train. Motorcycle crashes are a public health problem in Brazil and 13 adolescents died this way (Figure 4).

Regarding times of death, 51% occurred at the scene, 4.7% during pre-hospital care, 25.6% occurred at the hospital within the first 24 hours after admission, and the remaining 18.7% of deaths occurred after 24 hours after admission to the hospital. Gun-related injuries carried a 49% mortality rate at the scene, followed by transport-related deaths (19%) and drowning (14%).

When we analyzed the deaths according to the intent, homicides occurred in 50.6% of cases and were more frequent in the 10-17 age group. Unintentional injuries occurred in 48.5% of deaths and traffic-related injuries were the most common. Self-inflicted injuries were identified in only 5 cases (0.9%).

## Discussion

Studies related to mortality are useful in order to develop preventive strategies. In the present study deaths from trauma-related causes were predominantly amongst males. Studies conducted in various countries (the USA, Qatar, South Africa, Brazil, Sweden, China and India) showed the same pattern of results [6,9,11-15]. The reasons for this dominance, according to some authors, are greater exposures of males to risk factors such as alcohol abuse, drugs, increased interest in, and easier access to, firearms and vehicles such as cars or motorcycles, in addition to a greater integration into the labor market via legal or illegal activities. Another male-related feature is their greater impulsive and inquisitive nature, and their activities are more greatly related to intense emotions and adventure [12,16,17].

Several studies have shown that the majority of deaths from external causes in children under 18 years of age occurred between the ages of 10 and 17 years, as also reported in the present series. However, the causes of injury differ depending on the socioeconomic level of each country or region [8-14,16,18]. Another study conducted in African countries in 2009 differs from the above mentioned studies. The authors identified the group of greater mortality as the 1-4 year age group, and lack of adequate care was directed linked to those deaths [15].

In our series, the most prevalent causes of injury were gun-related injuries, traffic-related events and drowning. Adjusting for the total population growth, it was clear that gun-related injuries have decreased over time, while traffic-related events showed a slight increase in the period 2005-2008.

Currently, violence is a major public concern in all societies, especially in underdeveloped or developing countries. Gun-related injuries in this study were more prevalent in the 15-17 age group. These results were consistent with studies carried in other regions of Brazil [6,8]. One explanation for this fact is related to how urbanization has been developed in this country. There has been a high rate of internal migration, mostly young people in search of new employment opportunities in the large urban centers. However, most of these young people have not been absorbed by the labor market, thereby increasing marginalization on the periphery of large cities. This concentration of population associated with lack of employment and personal frustration causes these young individuals to be exposed to different forms of violence [6,8].

In a recent U.S. study, conducted in 2008 by some of the present authors, in San Diego, California, it was shown that gunshot wounds were the third leading cause of death in children under 18 years of age [11]. In another Brazilian study, it was shown that the rate of violence-related death rates has increased almost five-fold during the period from 1979 to 1995 [6]. On the other hand, in some regions such as Qatar, South India, China and Canada, deaths from firearm-related injuries are uncommon [10,13,14,17].

Related to trauma-related injuries, the World Health Organization (WHO) considers traffic accidents as a major public health problem worldwide and that effective preventative measures are not taken, the trend is an overall increase of deaths with traffic accidents being the secondary cause [19]. This study shows that traffic accidents are a cause of death in all age groups, but the emphasis is on the > 10 year old age group. Literature data show that in most studies the main cause of deaths from trauma-related injuries in children under 18 years is related to traffic accidents [9,10,12-15].

Several studies have attempted to elucidate the risk factors related to deaths from traffic accidents [19-22]. There are human factors, such as driving under the influence of alcohol, stress and fatigue, and excessive speed and inexperience of young drivers. Factors related to the road system include poor road signs, bad road conditions such as poor surface maintenance and a lack of kerbs. Factors related to vehicles include inadequate tire, brake and engine maintenance and a lack of efficient airbags.

Specifically in relation to traffic accidents, this study demonstrated that up to the age of 14 years, there were more cases of injuries to pedestrians, struck by vehicles, than to vehicle occupants. According to studies on African countries, the increased mobility of children in this age group, the fact that they are care-free and walk in groups, together with a lack of guidance, all justify a greater number of pedestrian accidents in this age group. The present study shows that in the 15-17 year age group, the frequency of deaths of pedestrians and vehicle occupants were similar. Studies show that in countries like Mexico and Colombia, accidents involving pedestrians are also more frequent [19,21]. This high frequency of accidents involving pedestrians can be related to the high influx of rural migrants to cities because they are not accustomed to the often chaotic traffic of the cities.

The present study revealed that 20% of deaths related to transport accidents were associated with motorcycles. In Brazil, the proportion of deaths related to motorcycle traffic rose from 4.1% in 1996 to 28.4% in 2007 [4]. Carrasco et al. [22] observed that the Campinas’ motorcycle fleet is growing four times faster than its population. In 2009, Campinas had 126% more motorcycles than in 2001, and between 2001 and 2009, 479 people died as consequence of motorcycle crashes in the city of Campinas. This type of problem was also observed in parts of Asia and India [12]. Despite the obvious advantages of cost (purchase price, fuel costs per mile and maintenance), many studies have shown that the high risk of fatality and injury is much higher in motorcycle accidents than in other categories of motor vehicles. The vulnerability of motorcyclists is higher and fatality in an accident is 14 times more likely compared to car occupants [22,23]. Despite the laws regulating the use of helmets, safety equipment and the practice of traffic safety most of these rules are blatantly ignored in Brazil by motorcycle drivers.

The cause of death described as drowning is also described as an important cause of death in literature [11,15]. In this series there was a large number of drowning incidents among 1-4 year olds, and another peak among 10-17 year olds. The deaths in the younger age group may be due to negligence or absence of preventive measures such as grids or screens around pools. In a study from India evaluating deaths in children under 5 years, drowning was the first cause. In the 10-17 age group, these deaths are more common in boys, usually engaged in work activities or recreation near ponds or rivers [15]. Another study conducted in China indicates that the majority of these accidents occur in rural areas [13].

Approximately 50% of deaths in this study occurred at accident scenes, and most of them were due to gunshot wounds. These data are consistent with a study conducted in another region in the state of São Paulo and in several American cities such as Los Angeles, San Francisco and Vermont [24,25]. In another American series, in Colorado, we found that most deaths occurring in less than 24 hours were due to traffic accidents [26].

Regarding intent, this study showed that the primary cause of death was homicide (50.6%), followed by accident (48.5%) and much lower, suicide (0.9%). These data are extremely alarming when considering the growing violence in our society and the social and economic repercussions that this may cause. The same pattern of intent was described in a study conducted in Recife, in the state of Pernambuco, and in another U.S. study conducted in Denver [6,27]. Other studies in Canada, Nepal, South Africa and China show accidents as the leading cause of death in children and adolescents [10,13,28,29]. It is interesting to note that a study in India, relating to the period of 1994 to 2005, showed that there were no cases of homicide in adolescents under 19 years of age [12].

In relation to suicide, this is an emerging problem in developed countries. In the U.S.A., it is the second most common cause of death in children in the 10-14 year age group and in a study conducted in Sweden in 2002, it was the first cause of death among 5-25 year olds [9,12].

Undisputed is the association between violence and alcohol misuse, illicit drug use and availability of firearms [4]. Other factors also related to homicide in younger children were described by Fujiwara et al. [30] in a study conducted in 2009, which used data from the National Violent Injury Statistics System in the U.S.A. The study indicated that the main victims of homicide aged less than 2 years were boys, whose parents had depression and financial problems [30].

The first measure in reducing deaths from trauma-related causes is prevention. These measures are specific to the host, the cause of death and the environmental and social factors surrounding the problem. Much of what has already achieved success in relation to prevention has been linked to active prevention associated with a mix of laws, educational programs and focuses on multidisciplinary and well-distributed teams, as well as the strengthening and organization of the state. In Brazil there are different initiatives bringing together the efforts of Federal, State and Municipal Governments and civil society aimed at addressing violence in general, and specifically among young people [4]. In 2003, the National Congress passed a law known as the Disarmament Statute, ruling on the registration, possession, and commercialization of firearms. In 2004 the government created the National Public Security Force to address urban violence and reinforce the state’s presence in regions with high-crime rates [4]. These actions help to explain why gun-related homicides have been trending downward since 2004.

Several studies focused on the prevention of accidents have shown a decrease in the number of deaths, through actions such as the use of smoke detectors, containment systems specifically for children in transport (car seats), use of helmets, protective netting on windows, hedges or fences around swimming pools, and specific laws related to speed limits, zero tolerance to drinking and driving, among other measures [31-34].

This study has the limitation that the deaths occurring in Campinas cannot express the true situation in Brazil, a country with various social disparities. Another limitation is that this epidemiological study considered only deaths, the majority occurring at the scene, and this is not enough to guide prevention programs, since the pediatric trauma population admitted to the hospital is different, mainly according to the cause of trauma. Baracat et al. [35] studying 3,214 children (less than 14 years old) in trauma-related accidents admitted to our university hospital in 1997/1998 observed: males predominated (62.1%); injuries were more common in the 9-13 year age group (33.4%) and 2-5 year age group (27.2%); fall was the cause in 74% of cases, and 89.7% of admissions were of low complexity.

## Conclusions

We conclude that among children and adolescents, there is a predominance of deaths arising from trauma-related injuries amongst males aged 14-17 years, mainly from gunshots and with homicide as the main intention. The gun-related deaths have decreased since 2004. These findings are useful in guiding further development and implementation of intervention measures and prevention strategies in this municipality in order to reduce deaths from trauma-related injuries in children and adolescents.

## Competing interests

The authors declare that they have no competing interests.

## Authors’ contributions

AMF and JB-S participated in the conception, design and intellectual content, literature review, collection, analysis and interpretation of data. TMF and GPF contributed to the medical records, literature review and manuscript writing. MCR and ECB contributed to the statistical analysis and manuscript writing. RC contributed to the conception, design, intellectual content, and manuscript writing. All authors read and approved the final manuscript.
